# Health impacts of household energy use:  indicators of exposure to air pollution and other risks

**DOI:** 10.2471/BLT.14.144923

**Published:** 2015-05-05

**Authors:** Kendra N Williams, Amanda L Northcross, Jay P Graham

**Affiliations:** a Department of International Health, Johns Hopkins Bloomberg School of Public Health, Baltimore, United States of America (USA).; b Department of Environmental and Occupational Health and Department of Global Health, George Washington University School of Public Health and Health Services, Washington, DC, USA.

The information gained from national surveys is essential for monitoring performance towards health goals and targets and informing allocation of resources for health priorities. Recent evidence of the negative impact of household air pollution on health suggests that it is time to upgrade national surveys to inform decision-making on improved fuels and cookstoves.

More than 40% of the world’s population rely on solid fuels such as wood, crop residues or dung for their cooking and heating needs.^1^ Household air pollution, caused by cooking indoors with solid fuels, is the third leading risk factor for morbidity and mortality globally. In 2010, 3.5 million deaths and 4.3% of global disability adjusted life years were attributable to household air pollution.^2^^,^^3^ Pollutants from inefficient combustion of solid fuels, especially black carbon particles, also contribute to global climate change.^1^^,^^4^

Consistent and correct use of improved cookstoves and cleaner fuels have the potential to improve health, reduce deforestation, mitigate climate change and improve livelihoods.^4^ Cleaner fuels such as liquefied petroleum gas or ethanol may be required to reduce exposures for critical health gains, but improved solid fuel stoves currently remain the only widely available and affordable option in many regions of the world.^4^ Although studies have shown that near exclusive use of an improved stove may be necessary to achieve measurable health benefits, evidence on the health and other benefits associated with improved stoves is growing.^5^^,^^6^

The Demographic and Health Survey (DHS) and the Multiple Indicator Cluster Survey (MICS) are two nationally-representative household surveys that have been widely used to collect data on health risks and outcomes since 1984 and 1995, respectively.^7^ To date, surveys of this type have been conducted nearly 600 times in roughly 190 countries, typically every three to five years among 5000 to 30 000 households.^8^^,^^9^ These surveys collect information using a standard model questionnaire approach to produce data that are comparable within and across countries over time.^7^ The sample is representative at national and regional levels and for urban and rural residence. Sampling is based on a stratified two-stage cluster design.^8^ The topics included in national surveys have evolved over time based on the data needed by host countries.^7^ Among approximately 400 questions in MICS and 850 in DHS, only three questions in both, plus two additional questions in MICS, are used to assess household air pollution.^8^^,^^9^ These five questions ask about the type of fuel used for cooking and where cooking takes place (Box 1). The type of cooking fuel is used as a proxy measure for estimating exposure to emissions from cooking; fuel used for heating or lighting is not included. Accurate estimates of personal exposure to household air pollution are not possible based on this information.^1^ The location of the stove (e.g. indoors or outdoors) provides insight into probable exposure levels for household members. However, this indicator lacks sufficient detail to determine specific exposure levels for household members. Only the MICS has two questions on whether a child collected firewood (or water) for household use and how much time was spent on these activities.

Box 1 **Survey questions used as indicators in the household energy sector ^8^^,^^9^**

**Indicators in Demographic and Health Surveys and Multiple Indicator Cluster Surveys**

- What type of fuel does your household mainly use for cooking?
- Is the cooking usually done in the house, in a separate building, or outdoors?
- Do you have a separate room that is used as a kitchen?

**Indicators in Multiple Indicator Cluster Surveys**

- During the past week, did (name) fetch water or collect firewood for household use?
- Since last (day of the week), about how many hours did he/she fetch water or collect firewood for household use?

More information could be collected through national surveys to increase awareness and knowledge of the extent and impact of household air pollution. Because the development of specific questions for national surveys is a lengthy process that requires extensive piloting and testing to assure validity and reliability, we do not propose specific questions, but rather summarize categories of information critical for understanding the problem, based on examples from a related field: water, sanitation and hygiene (WASH).

In contrast to the WASH field, no indicator in the DHS or MICS tracks types of cooking apparatus owned or used for cooking. Information on fuel collection is also incomplete. We suggest that additional indicators are needed in the following categories: (i) types of cooking apparatus owned; (ii) use of cooking apparatus; (iii) fuel collection practices; (iv) fine particulate matter exposures or household concentrations; and (v) fuels used for heating and lighting.

## Cooking apparatus

Because households often use multiple stoves or open fires for cooking, national surveys should collect data on different types of cooking apparatus owned by households to determine the extent of this practice.^10^ Documenting how often each apparatus is used will also be critical to monitoring progress.^10^ This information would help to identify areas where clean cooking technologies and fuels may be available but are not in regular use. Use of a cookstove classification system and stratification by fuel type could facilitate trend analyses within and across countries, as well as identify subpopulations to be targeted for interventions.^11^

New indicators could therefore address: (i) types of cooking apparatus and fuels used for cooking and (ii) time spent cooking with each cooking apparatus and fuel combination.

## Fuel collection practices

Researchers have noted that when fuel is collected rather than purchased, women and children are the major gatherers.^12^ The negative impact of water collection is well documented, but more evidence is needed to determine the impact of fuel collection, considering time and energy demands and potential exposure to violence and injury.^13^ Indicators on fuel collection would identify who typically collects solid fuel and how fuel collection may affect educational, health and development outcomes.

Indicators could include the following categories: (i) fuel source – bought or collected; (ii) primary fuel collector; (iii) fuel collection time; (iv) distance between household and fuel source; (v) number of fuel collection trips per week; and (vi) typical fuel loads carried.

## Air pollution exposure

Exposure to household air pollution is not assessed in any national health survey and current estimates on global exposure are based on fuel type as a proxy indicator.^3^ Measurement of actual exposures could facilitate identification of the most at-risk populations for interventions. Monitoring representative sub-samples of households using low-cost particulate matter samplers would result in more accurate estimates of exposures to household air pollution and subsequent estimates of the health impacts.

Fine particulate matter (PM_2.5_ – consisting of particles less than 2.5 μm in diameter) is a widely used indicator of air pollution exposure. Although no solid fuel stove has yet resulted in indoor air pollution concentrations that meet World Health Organization (WHO) guidelines, measuring PM_2.5_ could improve understanding of which technologies and fuels are truly effective at reducing exposures.^6^ The cost and complexity of collecting accurate measurements that are representative of human exposures will need to be weighed against the information gained.

## Fuels for other purposes

Currently, DHS and MICS do not gather information on the type of fuel used for heating or lighting. These practices can significantly affect air pollution exposure, especially the use of kerosene, which emits many health-damaging pollutants.^14^ A potential new indicator would include the type(s) of fuel used for purposes other than cooking.

## Discussion

Given the significant burden of disease and environmental impacts associated with cooking with unimproved stoves and fuels, more effort is needed to identify indicators that will accelerate progress. Because adoption of improved cooking technologies is currently the primary option for large sectors of the population, indicators are needed to effectively track changes in the ownership and use of select cooking technologies.^4^ Indicators on fuel collection practices are essential to understand potentially damaging effects on health, development and the environment.

The benefits and costs of any proposed indicator will need careful consideration. We have not assessed the resources needed for designing significant changes to national surveys, which may result in changes to sampling schemes and the workload of interviewers. We recognize that economic constraints could hinder data collection and research would be needed to understand the time and resources required.

The morbidity and mortality linked to cooking with solid fuels are significant, with particular implications for women and children. The impetus for assessing new indicators is motivated by a need to more fully understand how the household energy sector is changing in low- and middle-income countries. We recommend that new indicators be developed and rigorously evaluated to ensure that the value and practicality of current surveys is not diminished. The information gained from improved indicators has the potential to better inform the targeting of resources and design of strategies for reducing household air pollution.
